# Effect of Sexual Partnerships on Zika Virus Transmission in Virus-Endemic Region, Northeast Brazil

**DOI:** 10.3201/eid3012.231733

**Published:** 2024-12

**Authors:** Tereza Magalhaes, Flávio Codeço Coelho, Wayner V. Souza, Isabelle F.T. Viana, Thomas Jaenisch, Ernesto T.A. Marques, Brian D. Foy, Cynthia Braga

**Affiliations:** Texas A&M University, College Station, Texas, USA (T. Magalhaes); Fundação Getulio Vargas, Rio de Janeiro, Brazil (F.C. Coelho); Instituto Aggeu Magalhães-Fundação Oswaldo Cruz, Recife, Brazil (W.V. Souza, I.F.T. Viana, E.T.A. Marques, C. Braga); Colorado School of Public Health, Aurora, Colorado, USA (T. Jaenisch); Heidelberg University Hospital, Heidelberg, Germany (T. Jaenisch); University of Pittsburgh, Pittsburgh, Pennsylvania, USA (E.T.A. Marques); Colorado State University, Fort Collins, Colorado, USA (B.D. Foy)

**Keywords:** Zika virus, ZIKV, arbovirus, arboviral disease, dengue virus, chikungunya virus, CHIKV, sexual transmission, vector transmission, modeling, gender inequality, sexually-transmitted infections, vector-borne infections, viruses, zoonoses, Brazil

## Abstract

The epidemiologic effects of Zika virus (ZIKV) sexual transmission in virus-endemic countries remain unclear. We conducted a 2-level, linear mixed-effects logistic regression analysis by using a recently acquired population-based ZIKV and chikungunya virus (CHIKV) serologic dataset obtained from persons residing in Northeast Brazil (n = 2,070 participants). We adjusted mathematical models for housing type and age of participants; the models indicated a significantly higher likelihood of ZIKV seropositivity among persons engaged in a sexual relationship within the same household (odds ratio 1.25 [95% CI 1.00–1.55]; p = 0.047), regardless of their partner’s ZIKV serostatus, and among participants with a ZIKV-seropositive sex partner within the same household (odds ratio 1.54 [95% CI 1.18–2.01]; p = 0.002). CHIKV was also modeled as a control; no sex-associated effects were observed for CHIKV serology. Inclusion of ZIKV sexual transmission in prevention and control strategies is urgently needed, particularly in ZIKV-endemic regions.

In the mid-2010s, Zika virus (ZIKV) emerged as a global health threat despite its discovery in Uganda >6 decades earlier. After spreading across the islands of the South Pacific, the virus reached the Americas in 2013–2014, rapidly infecting >100 million persons during a 2015–2017 pandemic (*1*). The pandemic was a source for new information on ZIKV pathogenesis, including its potential to induce severe neurologic conditions, such as Guillain-Barré syndrome and congenital Zika syndrome (CZS) (*2*). It has been hypothesized that the primary mode of ZIKV transmission is through the bite of infected *Aedes* spp. mosquitoes (vector transmission). However, the virus can also be transmitted from mother to fetus through the placenta, potentially leading to CZS, and between persons via sexual intercourse (*2*,*3*).

Although ZIKV sexual transmission is recognized by health authorities and the scientific community, determining its epidemiologic relevance and how it contributed to the explosive spread of the virus has proved challenging. The main difficulty lies in distinguishing whether ZIKV infection occurred through mosquito bites or sexual contact, especially in affected persons living in countries with intense mosquito transmission of the virus.

Three field studies have shed light on ZIKV sexual transmission in regions highly affected by the 2015–2017 ZIKV pandemic. The first study was conducted in Puerto Rico and included participants acutely infected with ZIKV and their household members (*4*). The findings indicated that pairs of persons within the study households engaging in sexual relationships had a higher risk for both persons being ZIKV positive by PCR than other household pairs. The second study, conducted in Northeast Brazil by our group (T.M., T.J., E.T.A.M, and B.D.F.) (*5*), used a household-based serosurvey and showed that, within households, persons reporting sexual relationships with ZIKV-seropositive index-participants were 3 times more likely to also be ZIKV-seropositive than those without a sexual relationship with the index person. In addition, persons belonging to a sex dyad within households were more likely to be ZIKV seroconcordant than pairs who had no sexual relationship. The same serologic analysis was performed for chikungunya virus (CHIKV) and no associations between paired serologic data and sexual activity among the study participants were observed (*5*). That serosurvey recruited persons who had experienced acute febrile illness caused by arbovirus infection a few months before recruitment (thus, not acutely infected) along with their household members. The third study, also conducted in Northeast Brazil, reported that men residing in a low socioeconomic area and engaging in risky sexual behaviors were more likely to be seropositive for ZIKV than men who did not report risky sexual behaviors (*6*). Those 3 studies provide valuable data supporting sexual activity as a risk factor for ZIKV infection in countries that have intense mosquito transmission; however, factors, such as selection bias (e.g., choosing households on the basis of persons with symptomatic disease) (*5*) and relatively low numbers of participants (*4*–*6*), limit the generalizability of those findings.

Mathematical models have also been used to estimate the contribution of ZIKV sexual transmission in Zika epidemiology in regions affected by epidemics. In most cases, studies used national datasets of notified ZIKV cases in affected countries, such as Brazil and Colombia. Although some models have indicated minimal contribution of sexual transmission to ZIKV epidemics (*7*–*9*), the models that considered the synergistic effects of both mosquito and sexual transmission and heterogeneity of sexual relationships have indicated a more substantial role for sex in ZIKV infections overall (*10*–*13*). However, studies relying on official case notifications might be biased for several reasons. In particular, notified cases do not accurately represent the actual number of affected persons because of underreporting or misdiagnosis. Moreover, biases in gender-based healthcare seeking need to be considered when interpreting data from official notified cases.

We conducted a population- and household-based ZIKV seroprevalence study in an urban center in Northeast Brazil ≈3 years after ZIKV was introduced in the region (*14*); that serosurvey assessed past exposure to ZIKV and other arboviruses among residents of an arbovirus-hyperendemic region, ensuring representative demographic, socioeconomic, and spatial coverage. Using a large dataset obtained from that study (*14*), we performed a secondary analysis of the effect of sexual relationships on ZIKV transmission. The Institutional Review Board of Instituto Aggeu Magalhães approved the original study (protocol/Certificado de Apresentação de Apreciação Ética no. 79605717.9.0000.5190). We used the existing dataset and remained within the scope of the original study aims; thus, no additional approval for the secondary data analysis was required.

## Methods

### Primary Data Source

We conducted a stratified multistage cluster sampling survey to estimate the seroprevalence of ZIKV, CHIKV, and dengue virus (DENV) in residents of the city of Recife, Brazil, who were 5–65 years of age during August 2018–February 2019 (*14*). Recife, the capital of the state of Pernambuco, has an area of 218.8 km^2^ and a population of ≈1.5 million persons (*15*). Successive arbovirus outbreaks have been registered in the city since the introduction of DENV in the 1980s (*16*–*18*). After the surge of ZIKV cases in Brazil in 2015, Recife was a hotspot for microcephaly cases in babies associated with ZIKV infection (*19*).

The methods used for the original serosurvey have been previously described (*14*). In brief, we divided the population sample into high, intermediate, and low socioeconomic strata. We used a 2-stage sampling approach involving the random selection of census tracts followed by selection of households. All residents who were within the study age range (5–65 years) in the selected households were eligible to participate in the survey. Of the 2,691 eligible participants, we obtained ZIKV, DENV, and CHIKV serologic data for 2,070 persons; 480 persons were in the high, 815 in the intermediate, and 775 in the low socioeconomic stratum. We included a total of 899 households in the study. We collected individual and household data through interviews performed during home visits by using standardized questionnaires. In addition to collecting sociodemographic data and documenting clinical manifestations of arbovirus infections, we questioned participants about having had a fixed sex partner within the past 4 years. If the response was affirmative, they were then asked whether the sex partner resided in the same household. If the sex partner lived in the same house, study participants were prompted to specify which resident was their partner. After the interview, we collected a venous blood sample from each participant.

We used commercially available or in-house ELISAs to detect ZIKV and CHIKV immunoglobulins (IgG, IgG3, and IgM) in serum samples, as previously described (*14*). Assay sensitivities and specificities and IgG, IgG3, and IgM seroprevalence rates were determined as previously described (*14*). In addition, a subset of randomly selected serum samples was assayed by using a plaque reduction neutralization assay to further validate ZIKV IgG data. 

We used raw serologic data for ZIKV (IgG and IgG3) and CHIKV (IgG and IgM) and the associated variables of interest obtained in the serosurvey (*14*) for the analyses described in this work. Because the DENV seroprevalence rate found in the survey was high, which was expected, we did not include DENV data in the analyses.

### Data Analysis

We modeled the odds of testing positive for ZIKV in response to risk factors related to vector and sexual transmission by using a hierarchical 2-level linear mixed effects logistic regression (Appendix Figure). We considered having a sex partner (sexual transmission risk) and living with other ZIKV-seropositive participants (vector transmission risk) in the household as risk factors. Households formed the random intercept. We defined the binomial response variable by combining 2 diagnostic methods for each virus (tests for ZIKV IgG, ZIKV IgG3, CHIKV IgG, and CHIKV IgM) and assigned a positive status to persons who tested positive in >1 of the tests. We also investigated the effect of the residence type by adding another binary variable; a value of 1 was given for a person living in ground-level housing and 0 for a person living in a multistory apartment building. We included a person’s age in years in the model as a discrete variable. We calculated the odds ratio (OR) for age, which reflected the change in risk associated with each additional year of age. As a control, we used a model with the same structure to assess the odds of testing positive for CHIKV using the participants’ CHIKV serostatus. We reported ORs and 95% CIs for all risk factors included in the model. We interpreted OR values >1 as an increased probability of testing positive for ZIKV or CHIKV for each specific risk factor. We calculated p values according to a null hypothesis of OR = 1 and set the statistical significance threshold at 0.05. We estimated the models by using the lme4 package in R (The R Project for Statistical Computing, https://www.r-project.org).

## Results

Among the 2,070 study participants that had ZIKV and CHIKV serologic data, 1,207 (58.3%) were women and 863 (41.7%) men. We determined the frequency distribution of the main characteristics of the study population categorized by sex partner status (Table 1). Of the 2,070 participants, 891 reported having a sex partner within the same household; 873 (98.0%) of those reported being heterosexual, and 18 (2.0%) homosexual (Table 1). Only 7 persons who reported having a sex partner within the household were <18 years of age; each was 17 years of age. ZIKV and CHIKV serostatus data were missing for 211 partners of the 891 participants because of either refusal to have blood drawn or because they were absent during the home visit. As a result, those persons were excluded from the models that accounted for the sex partner’s ZIKV and CHIKV serostatus. In the other models, all 2,070 participants were included.

**Table 1:** Characteristics of survey participants according to sexual partnership status in study of ZIKV transmission in virus-endemic region, Northeast Brazil*

Characteristics	No. (%) study participants
Participants with a fixed sex partner within the household	891
Housing type†	
Ground-level house	638 (71.6)
Multistory apartment	250 (28.4)
Age group, y	
<18	7 (0.8)
>18	884 (99.2)
ZIKV serostatus of sex pairs‡	
Both partners positive	245 (27.5)
Discordant, 1 partner positive, 1 negative	256 (28.7)
Both partners negative	179 (20.1)
CHIKV serostatus of sex pairs‡	
Both partners positive	130 (14.6)
Discordant, 1 partner positive, 1 negative	215 (24.1)
Both partners negative	335 (37.6)
Participants with no sex partner within the household	1,179
Housing type	
Ground-level house	886 (75.4)
Multistory apartment	289 (24.6)
Age group, y	
<18	392 (33.4)
>18	787 (66.6)

*CHIKV, chikungunya virus; ZIKV, Zika virus. †Housing type was missing for 3 persons. ‡Data on sexual partners was incomplete for 211 persons, leading to their exclusion from the analyses of the effect of sexual partnership when accounting for the partner’s ZIKV and CHIKV serostatus.

In the ZIKV model, living in the same household with other persons who were ZIKV seropositive, regardless of whether they were sex partners, contributed significantly to the odds of testing positive for ZIKV (OR 1.47 [95% CI 1.17–1.84]; p<0.05) compared with living in a household with only seronegative persons. Having a sex partner within the household, irrespective of the partner’s ZIKV serologic status, increased the odds of testing positive for ZIKV by 64% (OR 1.64 [95% CI 1.36–1.99]; p<0.0001) compared with participants who had no sex partner in the household. The odds of testing positive for ZIKV increased by 94% (OR 1.94 [95% CI 1.51–2.5]; p<0.0001) when the sex partner in the household was ZIKV-seropositive compared with participants who did not have a ZIKV-seropositive sex partner. In contrast, the odds of being ZIKV seropositive when the sex partner was seronegative for ZIKV was low, suggesting a potential protective effect, although this effect was not significant (OR 0.79 [95% CI 0.60–1.03]; p = 0.086).

The type of residence was significantly associated with the odds of testing positive for ZIKV; persons living in ground-level housing had ≈3 times higher odds of being seropositive than those living in a multistory apartment building (OR 2.94 [95% CI 2.25–3.84]; p<0.0001). In addition, we previously found that ZIKV seroprevalence differed among age groups, being lower in persons <15 years of age (*14*); therefore, we fitted a model to assess the effect of age on ZIKV seropositivity. The model showed a positive association between age and ZIKV exposure (OR 1.03 [95% CI 1.02–1.03]; p<0.0001), indicating the odds of testing positive for ZIKV increased by 3% with each additional year of age. Because of the significant effects of both housing type and age on ZIKV serostatus, we adjusted for those variables and reanalyzed the effects of living with other seropositive persons, having a sex partner in the household, or having a ZIKV-seropositive sex partner in the household as risk factors for ZIKV seropositivity. In adjusted models, sex partnerships within the household remained a significant risk factor for ZIKV exposure (Table 2). The heterogeneity of households accounted for 6% variation in the odds of being ZIKV seropositive after controlling for the factors included in the model.

**Table 2:** Associations between sex partnership status within households and ZIKV or CHIKV seropositivity in study of ZIKV transmission in virus-endemic region, Northeast Brazil*

Exposure variables	Odds ratio (95% CI)	p value
ZIKV		
Age†	1.03 (1.02–1.03)	<0.0001
Housing type, ground level versus multistory apartment‡	3.25 (2.54–4.12)	<0.0001
Living with >1 ZIKV-seropositive person§	1.46 (1.13–1.88)	0.003
Sex partner in the household§	1.25 (1.00–1.55)	0.047
ZIKV-seropositive sex partner in the household§	1.54 (1.18–2.01)	0.002
ZIKV-seronegative sex partner in the household§	0.70 (0.52–0.94)	0.018
CHIKV		
Age†	1.01 (1.00–1.01)	0.005
Housing type, ground level versus multistory apartment‡	4.67 (3.28–6.65)	<0.0001
Living with >1 CHIKV-seropositive person§	2.84 (2.24–3.60)	<0.0001
Sex partner in the household§	1.05 (0.80–1.36)	0.739
CHIKV-seropositive sex partner in the household§	1.21 (0.82–1.80)	0.343
CHIKV-seronegative sex partner in the household§	0.71 (0.51–0.98)	0.035

*Mixed-effects hierarchical linear regression model was used to determine associations. Data were from a population-based survey conducted in Northeast Brazil during 2018–2019 (*14*). Number of study participants was 2,070. CHIKV, chikungunya virus; ZIKV, Zika virus. †Adjusted for housing type. ‡Adjusted for age. §Adjusted for age and housing type.

We fitted a separate model using the same structure for CHIKV serologic data to serve as a control. In the CHIKV model, living with other CHIKV-seropositive persons, regardless of whether they were sex partners, contributed significantly to the odds of testing positive for CHIKV (OR 2.59 [95% CI 1.94–3.46]; p<0.0001). Having a sex partner within the household did not contribute significantly to the odds of testing positive for CHIKV (OR 1.13 [95% CI 0.94–1.36]; p = 0.2). Similarly, having a CHIKV-seropositive partner in the household did not significantly contribute to the odds of being CHIKV seropositive (OR 1.29 [95% CI 0.97–1.74]; p = 0.09). Having a CHIKV-seronegative partner decreased the odds of testing positive for CHIKV (OR 0.69 [95% CI 0.54–0.89]; p = 0.004). The risk factor outcomes remained consistent in the CHIKV models adjusted for housing type and age (Table 2).

## Discussion

The role of sexual transmission in ZIKV epidemiology remains poorly understood, especially in virus-endemic countries having high levels of vector transmission. In a previous index- and household-based serosurvey conducted in Recife, Northeast Brazil, we identified sexual relationships within households as a risk factor for ZIKV exposure (*5*). The most substantial effect of sexual activity in that study was observed when analyzing ZIKV-seropositive index cases, whereby the likelihood of being ZIKV seropositive was 3 times higher for sex partners of ZIKV-seropositive index persons than for non–sex partners of those index persons within a household. That sex effect was not observed for CHIKV, which is transmitted by the same vectors as ZIKV and circulated in the study region around the same time as ZIKV but is not known to be sexually transmitted. That work involved index participants who had experienced symptomatic arboviral disease a few months before enrollment, along with their household members (*5*). In this study, we assessed whether similar effects would be seen in a dataset from a recent large population-based serologic survey designed to ensure representative demographic, socioeconomic, and spatial coverage across a wider area within the same region (*14*). Apart from the differences in the datasets (population-based rather than analyzing previously symptomatic index cases), distinct types of models were used.

Residing in a household with a person who was ZIKV or CHIKV seropositive was significantly associated with an increased risk for exposure to the respective virus. This outcome was expected because it is known that populations of *Aedes aegypti* mosquitoes, the vector of ZIKV and CHIKV, tend to establish themselves within domestic and peridomestic environments in residential areas. After a mosquito population is established within those areas and virus circulates, all susceptible residents of a house would have the same risk for vector transmission of the virus, assuming random mosquito biting of the residents. To corroborate this assumption, in the stratified analysis according to housing type, we found that persons residing in ground-level housing were 3 times more likely to be ZIKV or CHIKV seropositive than were persons living in multistory apartments. Efficient household transmission of arboviruses in areas with ground-level housing has been documented (*5*,*20*–*24*) and points to those houses being more easily infested with *Ae. aegypti* mosquitoes, likely because of mosquito species behavior and their proximity to more open-air water containers that can serve as breeding sites.

A critical finding from this study was that, in the models adjusted for housing type and age, having an active sex partner within a household increased the risk for ZIKV exposure by 25% and having a ZIKV-seropositive sex partner increased risk by 54%. Those findings corroborated our previous data (*5*) that showed a more robust association between increased risk for ZIKV infection and the sex partner’s seropositivity, indicating a higher level of virus exposure through sex corresponds to a greater risk of infection. Because of the low number of homosexual pairs in the study population, we were unable to evaluate potential differential risks between heterosexual and homosexual pairs. The differences in risk factors between those groups should be evaluated in future studies.

Serosurveys are limited in their ability to determine the timing of virus infection because previous exposure (indicated by antibody detection) could have occurred at any timepoint. Moreover, establishing a causal link between ZIKV infection and sexual activity requires assessing the timing of both events. One study conducted in Puerto Rico (*4*) investigated persons acutely infected with ZIKV and their sexual activity in the days preceding infection; this type of study is less susceptible to timing bias, and, consequently, can indicate a more precise causal link between sexual activity and ZIKV infection, although still indirectly. The population in this study was naive to ZIKV and CHIKV until those viruses were introduced and rapidly spread in the region during 2015–2016. After those initial outbreaks, a sharp decline in ZIKV and CHIKV transmission was observed in the region (*25*). We conducted the serosurvey (*14*) when local transmission of both viruses was minimal, meaning the seropositive persons captured in that study were likely infected during the first transmission wave of ZIKV and CHIKV, thus limiting the timeframe of infection occurrence. The underlying assumption in this study was that participants with a fixed sex partner in the household over the 4 years preceding the serosurvey likely maintained regular sexual activity during the period of ZIKV circulation in the region.

Serosurveys can also be biased by cross-reactivity in serologic assays. In this study, cross-reaction between DENV and ZIKV antibodies could have led to false positives. To minimize this cross-reactivity, we adjusted the cutoff value for the ZIKV ELISA according to well-characterized serum samples; however, some level of cross-reactivity likely still occurred. The residual cross-reactivity between ZIKV and DENV IgG might have slightly diluted the association between sexual partnership and ZIKV exposure, and a more specific test might reveal a stronger association. As previously described (*5*), the association between sexual partnership within households and ZIKV exposure was stronger when we used serologic data from plaque reduction neutralization tests than when we used IgG data from ELISAs. However, a potential higher-than-expected waning of ZIKV antibodies over time (*26*–*29*) could have resulted in some false negatives.

The central message from the population-level data is that sexual activity significantly contributes to ZIKV transmission at a household level in virus-endemic regions. This finding is supported by previous index-case research (*5*) and 2 other independent studies (*4*,*6*), including 1 involving persons acutely infected with ZIKV (*4*). Our findings also suggest that sexual transmission acts synergistically with vector transmission in ZIKV-endemic regions, but whether household transmission is more often initiated by a vector that then leads to sexual transmission in the household or vice versa remains unknown. Ultimately, the finding that increased risk for ZIKV exposure can be caused by sexual activity challenges the current belief that sexual transmission has minimal effect in ZIKV epidemiology, and transmission models should be recalibrated accordingly. This finding also emphasizes the need for health authorities and the scientific community to recognize ZIKV infection as a potential sexually transmitted disease. We advocate for the urgent inclusion of this transmission mode in ZIKV prevention and control strategies, particularly in virus-endemic countries. Although health authorities such as the World Health Organization and US Centers for Disease Control and Prevention have released official recommendations to prevent ZIKV sexual transmission (*30*,*31*), those recommendations have been primarily focused on travel-associated infections and CZS. However, sexual transmission should be considered in the broader context of ZIKV epidemiology because ZIKV infection can lead to different symptoms in an infected person. In addition, symptoms and long-term sequelae related to ZIKV-induced urogenital tract infections are not well understood, nor is the potential relationship between sexual transmission and CZS; both aspects warrant further investigation. For CZS, we strongly suggest including sexual behavior variables in Zika cohort studies, particularly in those studies conducting multivariable regression analyses on risk factors for congenital anomalies.

In conclusion, ZIKV transmission is associated with sexual activity. It is essential to consider gender and socio-economic factors within the context of ZIKV sexual transmission to develop appropriate prevention and control strategies. This need is particularly critical because ≈50% of the global female population have limited autonomy in determining their sexual and reproductive health and rights, as reported by the United Nations Population Fund (*32*). The involvement of male partners in prevention and control activities, for example, should be highly encouraged. Existing ZIKV control programs in virus-endemic countries, such as Brazil, that only focus on vector transmission are already biased against women, contributing to gender inequalities (*33*). Special attention should be given to populations at high risk of acquiring sexually transmitted infections, such as sex workers, when designing prevention and control strategies.

Appendix Additional information for effect of sexual partnerships on Zika virus transmission in virus-endemic region, Northeast Brazil.
