# Supplementary material for: Effect of Sexual Partnerships on Zika Virus Transmission in Virus-Endemic Region, Northeast Brazil
Source: Emerg Infect Dis. 2024 Dec;30(12):2559–66. doi: 10.3201/eid3012.231733 (PMC11616633; doi:10.3201/eid3012.231733)
Supplement: Appendix — Additional information for effect of sexual partnerships on Zika virus transmission in virus-endemic region, Northeast Brazil. [file 23-1733-Techapp-s1.pdf]

Article DOI: https://doi.org/10.3201/eid3012.231733

EID cannot ensure accessibility for supplementary materials supplied by authors. Readers who have difficulty accessing supplementary content should contact the authors for assistance.

Effect of Sexual Partnerships on Zika Virus Transmission in Virus-Endemic Region, Northeast Brazil

Appendix

$$E\left[y_{ij}\right] = Logit^{-1}\left(\beta_0 D_s + \beta_1 I_{ij}(pos_{hh} > 0) + U_j\right)$$

Appendix Figure. Mathematical model to determine odds of testing positive for Zika virus (ZIKV) or chikungunya virus (CHIKV) in response to risk factors related to vector and sexual transmission by using a hierarchical 2-level linear mixed effects logistic regression. The binomial response variable y represents the results of the combined serologic tests applied (i.e., positivity for ZIKV IgG or IgG3 and for CHIKV IgG or IgM). A positive status was assigned to persons testing positive in ≥1 of the tests. In the model, yij is the log-odds of individual i in household j testing positive for ZIKV (or CHIKV). Ds is a binary variable representing whether the individual is part of sex dyad. $Iij(poshh$>0) is another binary variable representing the presence of other positives in the household (1 if other positives live in the same household). Finally, Uj is the intercept or household-specific random effect. Uj is normally distributed (mean 0.008 [SEM 0.051]).
